# Polycomb chromobox Cbx2 enhances antiviral innate immunity by promoting Jmjd3-mediated demethylation of H3K27 at the *Ifnb* promoter

**DOI:** 10.1007/s13238-018-0581-0

**Published:** 2018-10-24

**Authors:** Donghao Sun, Xuetao Cao, Chunmei Wang

**Affiliations:** Department of Immunology and Center for Immunotherapy, Institute of Basic Medical Sciences, Peking Union Medical College, Chinese Academy of Medical Sciences, Beijing, 100005 China

**Keywords:** Cbx2, Jmjd3, interferon beta, innate immunity

## Abstract

Polycomb chromobox (CBX) proteins regulate gene transcription by maintaining chromatin states, which guide a variety of biological processes. Now, epigenetic regulation of innate immune response is an emerging field. However, the role of CBX proteins in innate immunity remains unclear. We confirmed that the expression of CBX family proteins, especially Cbx2, was decreased in macrophages upon viral infection, and then we investigated the role of Cbx2 in the antiviral immune response. Silencing or knockdown of Cbx2 in macrophages inhibited virus-induced production of IFN-β. Furthermore, heterozygous Cbx2 knockout were susceptible to VSV challenge. Mechanistically, Cbx2 binds to and recruits Jmjd3 to the *Ifnb* promoter, leading to demethylation of H3K27me3 and increased transcription of IFN-β. Together, our study reveals a non-traditional function of a Cbx protein and adds new insight into the epigenetic regulation of antiviral innate immunity.

**Electronic supplementary material:**

The online version of this article (10.1007/s13238-018-0581-0) contains supplementary material, which is available to authorized users.

## Introduction

The innate immune response is an important determinant in the fight against virus infection, deploying pattern-recognition receptors (PRRs), such as the RIG-I-like receptor (RLR), to sense and respond to pathogens (Cao, 2016; Kawai and Akira, 2010). Upon binding to pathogen-associated molecular patterns (PAMPs), PRRs activate a series of signaling cascades, leading to the induction of type I interferons (IFNs) by nuclear factor kappa-light-chain-enhancer of activated B cells (NF-κB), interferon regulatory factor (IRF) 3/7 and/or activator protein-1 (AP-1) (Iwasaki and Pillai, 2014; Wu and Chen, 2014). Subsequently, IFNs induce the expression of interferon stimulated gene (ISGs) to restrict viral replication through the activation of the Janus kinase (JAK)-signal transducer and activator of transcription (STAT) pathway (McNab et al., 2015). Precise control of the innate immune response is critical for maintaining host immune homeostasis and ensuring proper viral clearance.

Epigenetic modifications such as DNA methylation, histone modifications, noncoding RNA regulation and chromatin remodeling have been shown to impact gene transcription. Epigenetic regulation in innate immunity has been studied extensively in recent years, yielding insights into the detailed molecular mechanisms that modulate the expression of inflammatory cytokines and type I interferons (Marcos-Villar et al., 2018; Yoshida et al., 2015; Zhang et al., 2015). For example, Tet2 recruits Hdac2 to specifically repress IL-6 by histone deacetylation, which resolves inflammation (Zhang et al., 2015). Dnmt3a promotes activation of TBK1 by maintaining high expression of the histone deacetylase HDAC9, leading to the production of type I interferons and antiviral innate immunity (Li et al., 2016). The lncRNA lnc-Lsm3b binds to RIG-I monomers and restricts RIG-I protein’s conformational shift, thereby terminating type I IFNs production (Jiang et al., 2018). However, the identification of other unknown epigenetic modifier and the underlying mechanisms for innate immunity remain to be further explored.

The Polycomb group (PcG) proteins, which are best characterized by two protein complexes (Polycomb repressive complex 1 [PRC1] and Polycomb repressive complex 2 [PRC2] have been identified as critical regulators of gene expression that maintain repressive chromatin states (Bernstein et al., 2006; Simon and Kingston, 2013). Chromobox (CBX) family proteins are canonical components of PRC1 and are divided into two groups: HP1α(Cbx5), HP1β(Cbx1), and HP1γ(Cbx3); and Cbx2, Cbx4, Cbx6, Cbx7 and Cbx8. The latter group has an N-terminal chromodomain that serves as a recognition site and binds to H3K27me3, which helps to recruit and stabilize PRC1 to specific regions of the chromatin (Aranda et al., 2015; Di Croce and Helin, 2013). Accumulating evidence has demonstrated that CBX proteins play crucial roles in a variety of biological processes, such as the differentiation of stem cells, neurite development and tumor angiogenesis (Chen et al., 2017; Santanach et al., 2017; Sparmann and van Lohuizen, 2006). Recently, Cbx7 has been shown to repress activation-induced T cell apoptosis, indicating a role in the adaptive immune response (Li et al., 2014a, b). However, the role of CBX family proteins in innate immunity remains largely unknown.

In this report, we addressed the function of Cbx2 in antiviral innate immunity. Our results demonstrate that Cbx2 enhances the production of type I interferon by binding and recruiting Jmjd3 to the promoter of IFN-β, thus activating the antivirus immune response. Therefore, our work identifies Cbx2 as a positive mediator in antiviral immunity, which provides new mechanistic insight into epigenetic modifiers in the innate immune response.

## Results

### Cbx2 promotes virus-induced production of type I interferon in macrophages

**Figure 1:**
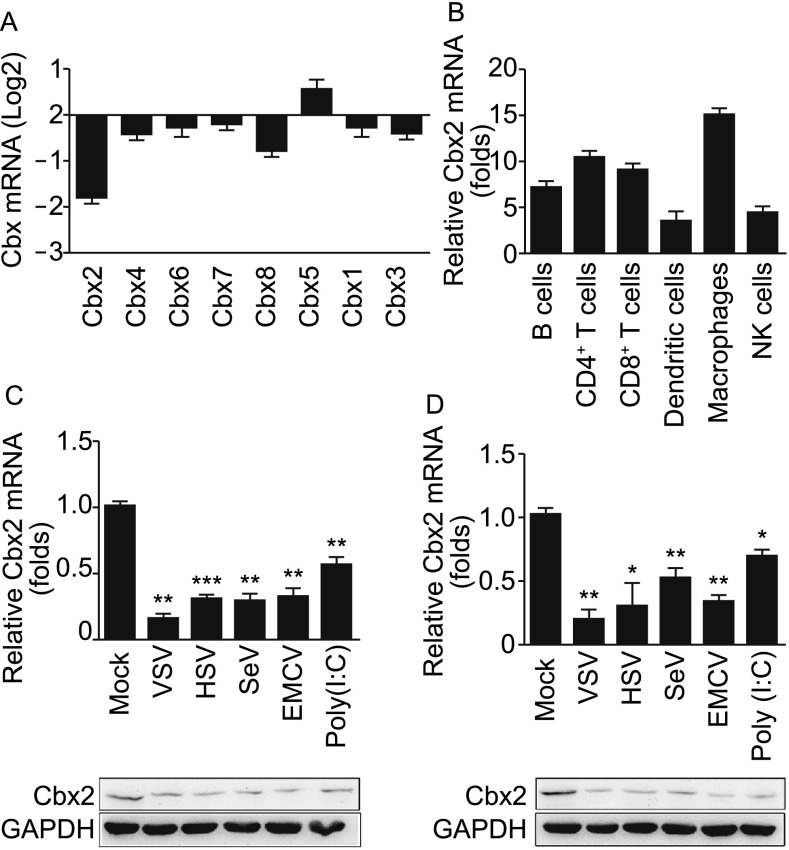
**Virus infection down-regulates Cbx2 expression in macrophages**. (A) Q-PCR analysis of CBX family proteins in mouse peritoneal macrophages infected with VSV (MOI = 10) for 8 h. The fold change was calculated relative to the expression in uninfected cells. (B) Q-PCR analysis of Cbx2 in different immune cells. NK, natural killer cells. Expression was normalized to the expression of β-actin. (C and D) Q-PCR analysis and Western blot of Cbx2 in mouse peritoneal macrophages (C) or RAW264.7 cells (D) infected with virus (MOI = 10) or incubated with Poly (I:C) (100 μg/mL) for 8 h. All data are from three independent experiments (mean ± SEM of technical triplicates) (C and D). Data are representative of three independent experiments with similar results. *P < 0.05; *P < 0.01; ***P < 0.001

Gene profiling data indicate the decreased expression of CBX family proteins is observed after infection with virus in the brain (GEO: GSE44331) and in RAW246.7 macrophages (GEO: GSE81675), inspiring us to investigate the role of CBX proteins in the innate immune response. We first examined the expression pattern of CBX proteins in macrophages upon virus infection. A reduction in the expression of CBX, and especially Cbx2, was observed in VSV-infected macrophages (Fig. 1A), suggesting that CBX proteins may be involved in the antiviral immune response. Because the expression of Cbx2 is highest in macrophages compared with other immune cells, such as T cells and natural killer cells (Fig. 1B), we focused on investigating the role of Cbx2 in antiviral innate immunity. A decrease in Cbx2 mRNA and protein were observed upon infection of mouse peritoneal macrophages (Fig. 1C) and RAW264.7 macrophages (Fig. 1D) with a variety of viruses in addition to VSV, and upon exposure to Poly (I:C), which suggested the decreased expression of Cbx2 might be involved in the antiviral innate immune response.

**Figure 2:**
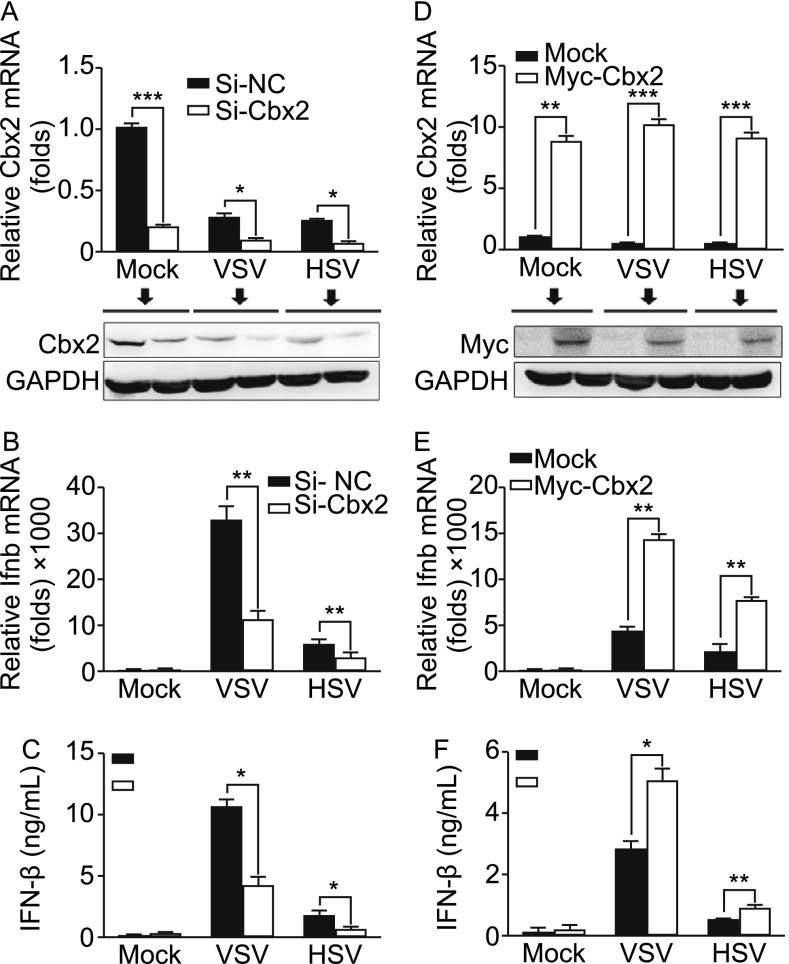
**Cbx2 promotes virus-induced type I interferon production in macrophages**. Q-PCR (upper panel) and Western blot (lower panel) of Cbx2 expression in mouse peritoneal macrophages. (B and C) Analysis of the level of IFN-β mRNA (B) and protein (C) in peritoneal macrophages with Cbx2 knockdown by Q-PCR and ELISA. Cells were transfected with a control siRNA (Si-NC) or a Cbx2-targeting siRNA (Si-Cbx2) for 48 h, and then infected with virus (MOI = 10) for 8 h (A–C). (D) Q-PCR (upper panel) and Western blot (lower panel) analysis showing effective overexpression of Cbx2 in Cbx2 stably over-expressing (Myc-Cbx2) RAW264.7 cells. (E and F) Q-PCR (E) and ELISA (F) analysis of virus-induced IFN-β in Mock or Myc-Cbx2 RAW264.7 cells infected with virus (MOI = 10) for 8 h. Cbx2 and IFN-β mRNA values were normalized to the expression of β-actin and all data are from three independent experiments (mean ± SEM of technical triplicates). Data are representative of three independent experiments with similar results. *P < 0.05; **P < 0.01; ***P < 0.001

We used small interfering RNA (si-Cbx2) to silence Cbx2 expression in mouse peritoneal macrophages that were then infected with VSV (RNA virus) or HSV (DNA virus) (Fig. 2A). A decrease in the mRNA level (Fig. 2B) and protein level (Fig. 2C) of IFN-β was observed in Cbx2 knockdown macrophages infected with virus, as well as IFN-α and IL-6, but not TNF-α (Fig. S1A and S1B). Consistently, overexpression of Cbx2 (Fig. 2D) enhanced VSV or HSV-induced production of IFN-β in RAW246.7 cells (Fig. 2E and 2F). These findings suggest that Cbx2 promotes antiviral innate immunity by enhancing the production of type I interferon.

### *In vivo* knockdown of Cbx2 increases susceptibility to VSV challenge

**Figure 3:**
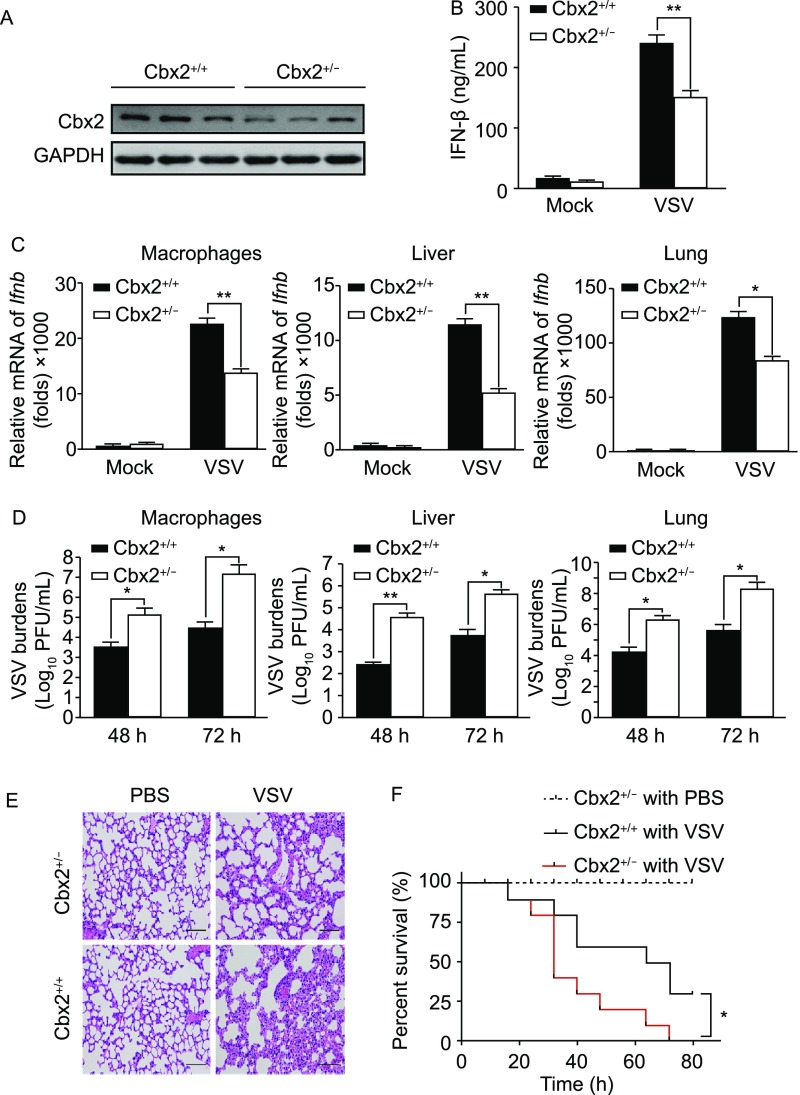
**Knockdown of Cbx2 increases susceptibility to VSV challenge**. (A) Western blot analysis of Cbx2 expression level in peritoneal macrophages derived from Cbx2^+/−^ mice as compared to Cbx2^+/+^ mice. (B) ELISA of IFN-β in serum of Cbx2^+/−^ mice and Cbx2^+/+^ mice (*n* = 6 per group) after challenge by tail vein injection (IOCV) of VSV (2 × 10^6^ PFU/g) for 18 h. (C) mRNA level of *Ifnb* in macrophages, livers and lungs derived from Cbx2^+/−^ mice or Cbx2^+/+^ mice after challenge with VSV (2 × 10^6^ PFU/g) for 18 h. (D) Plaque assay of VSV burden. Vero cells were infected at a MOI of 0.001 PFU/cell. Viral burdens were quantified by plaque assay in Vero cells after 48 h and 72 h and reported as log_10_ PFU/mL. (E) Hematoxylin and eosin staining of lung sections from Cbx2^+/−^ and Cbx2^+/+^ mice with or without challenge with VSV (2 × 10^6^ PFU/g) for 18 h. Scale bar, 100 μm. (F) Survival of Cbx2^+/−^ and Cbx2^+/+^ mice (*n* = 10 per group) after challenge with VSV (1 × 10^7^ PFU/g) for the indicated times. *P < 0.05 (Log rank test). The data in panels (B–D) is from three independent experiments (mean ± SEM of technical triplicates). All data are representative of three independent experiments with similar results. *P < 0.05; **P < 0.01

Because Cbx2 gene knockout is developmentally lethal (Katoh-Fukui et al., 1998), we used Cbx2 heterozygous knockout mice (Cbx2^+/−^ mice) to investigate the role of Cbx2 in the antiviral response *in vivo*. The *in vivo* reduction in Cbx2 expression levels in Cbx2^+/−^ mice was confirmed (Fig. 3A). When challenged with VSV, Cbx2^+/−^ mice produced significantly less IFN-β in serum than control mice did (Fig. 3B). Furthermore, the mRNA level of *Ifnb* was lower (Fig. 3C) and viral titers were higher (Fig. 3D) in macrophages, lungs and livers from Cbx2^+/−^ mice versus control mice. Additionally, more lung infiltration of inflammatory cells was observed in Cbx2^+/−^ mice than in control mice (Fig. 3E). Accordingly, Cbx2^+/−^ mice exhibited a shorter survival time when challenged with VSV (Fig. 3F). Therefore, our *in vivo* data further confirm that Cbx2 plays an important role in promoting antiviral innate immune responses.

### Cbx2 promotes the transcriptional activation of *Ifnb*

**Figure 4:**
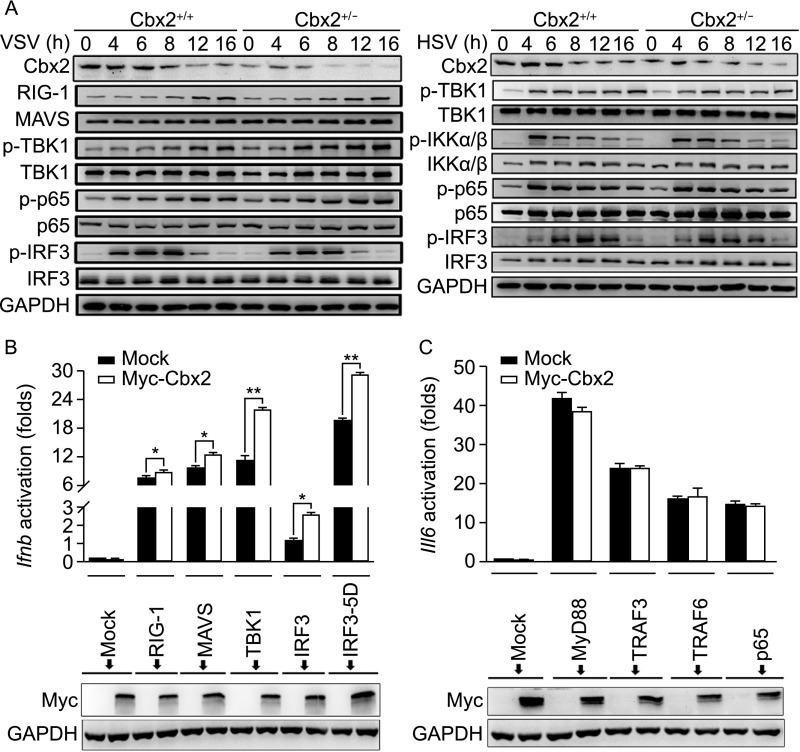
**Cbx2 enhances IFN-β transcription**. (A) Western blot analysis of mouse peritoneal macrophages derived from Cbx2^+/−^ or Cbx2^+/+^ mice after challenge with VSV or HSV (MOI = 10) for the indicated times. (B and C) Analysis of luciferase activity and myc-Cbx2 expression in lysates of HEK293T cells stably over-expressing IFN-β-Luc (B) or IL-6-Luc (C). Results were normalized by Renilla luciferase activity (*n* = 6). Data are representative of three independent experiments with similar results. All data are from three independent experiments (mean ± SEM of technical triplicates) (B). Data are representative of three independent experiments with similar results. *P < 0.05; **P < 0.01

To elucidate the underlying mechanism of Cbx2 in promoting the production of IFN-β, we examined the effect of Cbx2 on signaling molecules that are known to function within the viral response pathway, including proximal modulators (RIG-I, MAVS, TBK1, MyD88, TRAF3 and TRAF6) and key transcriptional factors (p65 and IRF3/IRF3-5D). The pathways were not significantly impacted by Cbx2-knockdown after infection with either VSV or HSV (Fig. 4A). We next examined the effect of Cbx2 on the activation of *Ifnb* promoter by known signaling activators. As shown in Figure 4B and 4C, Cbx2 significantly enhanced the luciferase activity in IFN-β-Luc-expressing cells but not in IL-6-Luc-expressing cells after transfection with proximal modulators and key transcriptional factors expression vectors. These results suggest that Cbx2 enhanced *Ifnb* transcriptional activation.

### Cbx2 enhances IFN-β activation by binding to Jmjd3

**Figure 5:**
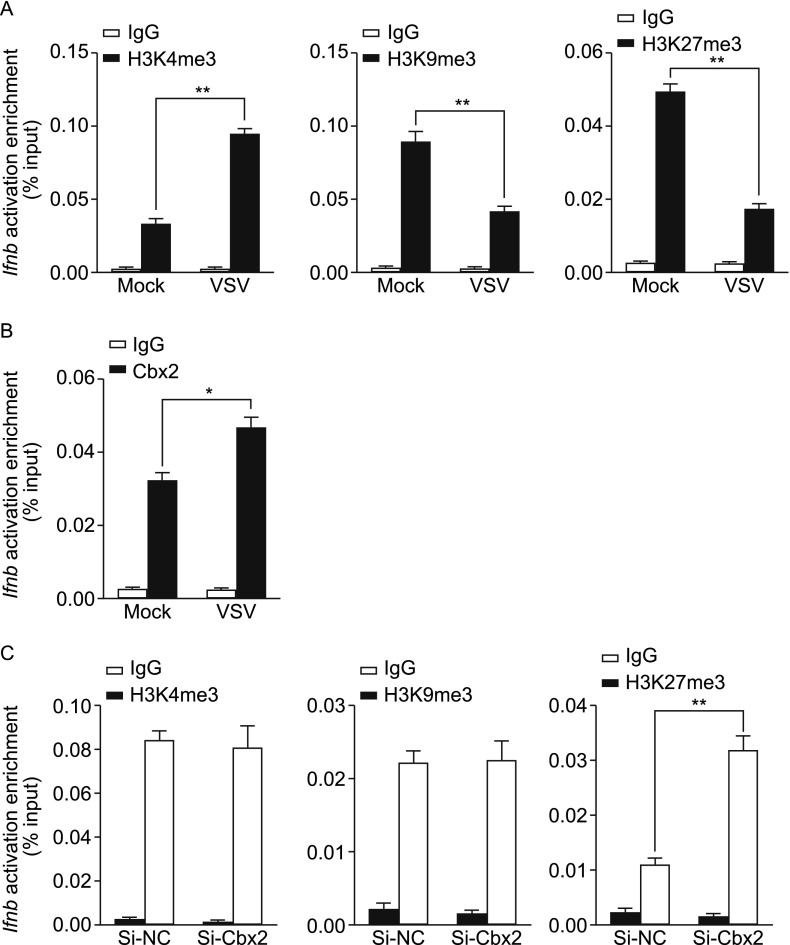
**Cbx2 suppresses the methylation3 of H3K27 on the** ***Ifnb*** **promoter**. (A) ChIP analysis of methylated histones at the *Ifnb* promoter in RAW264.7 cells left unstimulated (mock) or infected with VSV (MOI = 10) for 6 h. (B) ChIP analysis of Cbx2 at the *Ifnb* promoter in RAW264.7 cells left unstimulated (mock) or infected with VSV (MOI = 10) for 6 h. (C) ChIP analysis of methylated histones at the *Ifnb* promoter in mouse peritoneal macrophages transfected with Si-NC or Si-Cbx2 for 48 h, and then infected with VSV (MOI = 10) for 6 h. All data are from three independent experiments (mean ± SEM) of technical triplicates. Data are representative of three independent experiments with similar results. *P < 0.05; **P < 0.01

Because Cbx2 is a canonical component of PRC1, which is known to regulate gene expression epigenetically via chromatin modification, we assessed the role of histone modification in the transcriptional activation of IFN-β. As shown in Figure 5A, infection of macrophages with VSV caused a decrease in the recruitment of H3K27me3 and H3K9me3, and an increase in the recruitment of H3K4me3 to the *Ifnb* gene promoter. Furthermore, VSV infection caused an increase in the recruitment of Cbx2 to the *Ifnb* gene promoter (Fig. 5B). Silencing of Cbx2 increased the recruitment of H3K27me3 to the *Ifnb* gene promoter, but had no effect on recruitment of H3K9me3 or H3K4me3 (Fig. 5C). These results suggest that Cbx2 suppresses the methylation3 of H3K27 on the *Ifnb* gene promoter.

**Figure 6:**
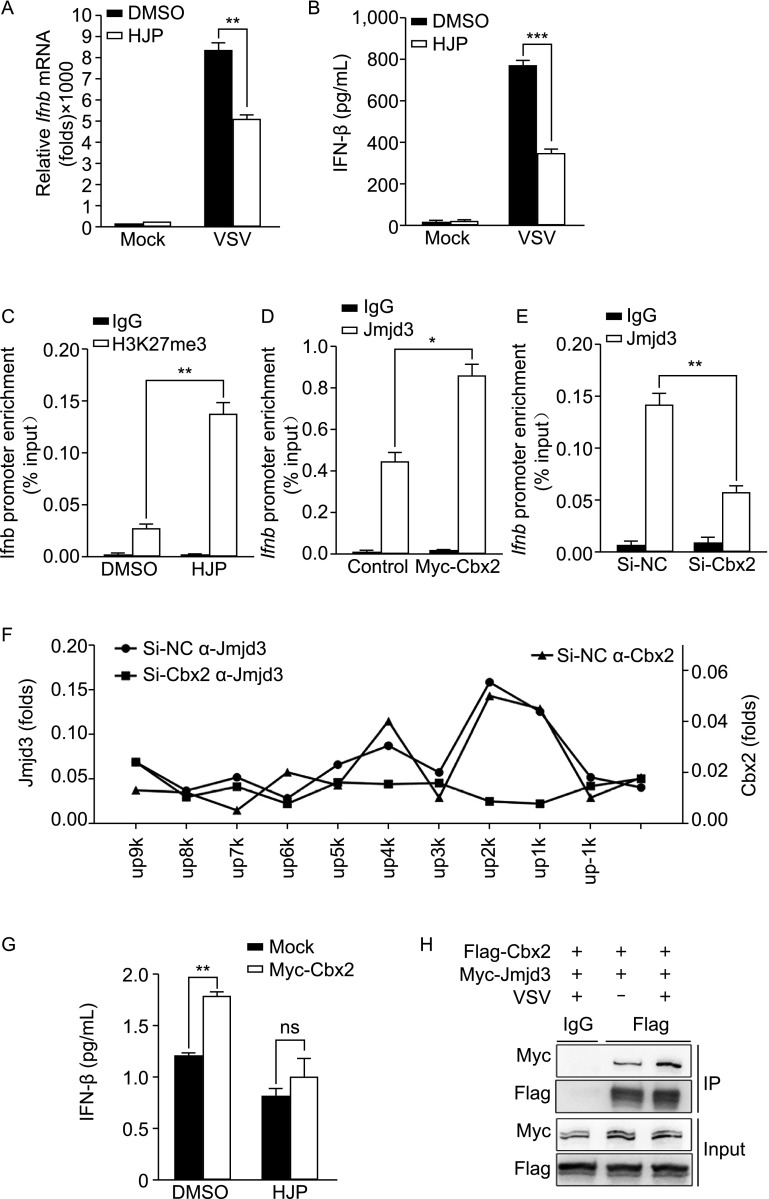
**Cbx2 enhances the activation of IFN-β activation by binding to Jmjd3**. (A) Q-PCR analysis of *Ifnb* expression, and (B) ELISA analysis of IFN-β in supernatants from mouse peritoneal macrophages treated with Jmjd3 inhibitor (HJP) for 6 h, and then infected with or without VSV (MOI = 10) for 6 h. (C) ChIP analysis of H3K27me3 at the *Ifnb* promoter in mouse peritoneal macrophages treated with HJP for 6 h, and then infected with VSV (MOI = 10) for 8 h. (D and E) ChIP analysis of Jmjd3 at the *Ifnb* promoter in Myc-Cbx2 RAW264.7 cells (D) or mouse peritoneal macrophages transfected with Si-NC or Si-Cbx2 (E). Cells were infected with VSV (MOI = 10) for 6 h. (F) ChIP analysis of Jmjd3 and Cbx2 at different regions of the *Ifnb* promoter in control or Cbx2-knockdown macrophages. (G) ELISA analysis of IFN-β in supernatants mock or Myc-Cbx2 RAW264.7 cells treated with or without with HJP for 6 h. (H) Western blot analysis of the interaction of Cbx2 and Jmjd3 in HEK293T cells transfected with Cbx2 and Jmjd3 expression plasmid. The proteins were immunoprecipitated with IgG or an anti-Flag antibody prior to immunoblotting. The data in panels (A–G) are from three independent experiments (mean ± SEM of technical triplicates). All data are representative of three independent experiments with similar results. *P < 0.05; **P < 0.01; ***P < 0.001

While our findings suggest that Cbx2 binding to the *Ifnb* promoter is associated with H3K27 demethylation, Cbx2 is not known to have activity as a demethylase *per se*. However, Jmjd3 is known to function as a specifically demethylase for H3K27 (Hong et al., 2007). Therefore, we speculated that Jmjd3 might be involved in Cbx2-mediated suppression of H3K27me3. To evaluate this possibility, we assessed the effect of a small molecule inhibitor of Jmjd3 (HJP). Indeed, Jmjd3-inhibitor decreased the mRNA (Fig. 6A) and protein production of IFN-β (Fig. 6B) after VSV infection and also increased the recruitment of H3K27me3 to the *Ifnb* gene promoter (Fig. 6C). Furthermore, overexpression of Cbx2 enhanced the recruitment of Jmjd3 to the *Ifnb* gene promoter (Fig. 6D); while conversely, knockdown of Cbx2 suppressed the recruitment of Jmjd3 to the *Ifnb* gene promoter (Fig. 6E), and this activity could be localized to the proximal 1 kb to 2 kb region (Fig. 6F). Moreover, inhibition of Jmjd3 abrogated Cbx2-mediated enhancement of IFN-β production (Fig. 6G). Lastly, we performed immunoprecipitation assays to show that Cbx2 directly bound to Jmjd3 (Fig. 6H).

Taken together, these data demonstrated that Cbx2 promotes antiviral innate response by binding to and recruiting Jmjd3 to the *Ifnb* promoter, leading to demethylation of H3K27me3 and increased transcription of IFN-β.

## Discussion

In this study, we identified that Cbx2 enhances virus-induced production of type I interferon, and we defined a molecular mechanism for this regulation in which Cbx2 binds to and recruits Jmjd3 to the *Ifnb* promoter, leading to demethylation of H3K27 and promotion of *Ifnb* transcription. Our findings identify a new epigenetic regulator with an integral role in the antiviral innate immune response, which suggests a noncanonical function of Cbx2.

CBX family proteins play crucial roles in a variety of biological processes by maintaining repressive chromatin states of specific genes. In tumors, CBX family proteins have been shown to endow PRC1 with distinct functions, such as oncogene or tumor suppressor functions. For example, Cbx7 has been identified to act as tumor suppressor in lung carcinoma by suppressing CCNE1 expression (Forzati et al., 2012); conversely, it has been shown to act as an oncogene in gastric cancer by repressing p16 (INK4a) (Zhang et al., 2010). Cbx2 family proteins have also been shown to inhibit neurite development by directly inhibiting neuron-specific gene expression. On the other hand, CBX family proteins also have been shown to enhance gene transcription in certain cases. For example, Cbx4 enhances the expression of VEGF and promotes angiogenesis in hepatocellular carcinoma cells (Li et al., 2014a, b), which is consistent with our findings that Cbx2 enhances antiviral innate immunity by promoting the transcriptional activation of IFN-β. Therefore, it is possible that these distinct functions of Cbx2 are cell-type-specific. In tumor cells, Cbx2 is up-regulated and has been identified as a potential drug target (Chen et al., 2017; Clermont et al., 2016), whereas our results demonstrate that Cbx2 is down-regulated in macrophages infected with virus. Therefore, we speculate that the distinct functions of Cbx2 might be explained both by cell-type-dependent function and expression differences. We also observed that other Cbx family proteins exhibit decreased expression in virus-infected macrophages, which raises the possibility that other Cbx family proteins might collaborate with Cbx2 in the antiviral response.

Conventionally, PRC1 proteins bind to H3K27me3 and recruit other components of PRC1 to chromatin by protein-protein interaction to further enhance transcriptional repression of target genes (Gao et al., 2012). However, it has been shown that certain CBX proteins can associate with moderators other than the PRC1 complex, displaying a PRC1-independent role in transcriptional regulation. For instance, Cbx4 represses the expression of Runx2 and metastasis of colorectal carcinoma, which is dependent on its interaction with HDAC3 (Wang et al., 2016). Furthermore, Cbx8 associates with WDR5, thereby enhancing Notch-related gene expression and promoting breast tumorigenesis (Chung et al., 2016). Intriguingly, our study suggests that Cbx2 enhances IFN-β transcription in virus-infected macrophages by inhibiting H3K27me3 of *Ifnb* promoter, which is dependent on the recruitment of Jmjd3. These findings suggest that the interaction with Jmjd3 is pivotal for the function of Cbx2 in antiviral innate immunity. An important issue for the current research is the spatiotemporal dynamics between Cbx2, Jmjd3 and H3K27me3. Furthermore, it would be important to investigate whether other moderator(s) of PRC may be involved in Cbx2-mediated promotion of IFN-β transcription.

In Cbx2^+/−^ mouse-derived macrophages, increased recruitment of H3K27me3 to the *Ifnb* gene promoter was also observed, suggesting that Cbx2 reduces H3K27me3 at the *Ifnb* gene promoter. This is in contrast to Polycomb group (PcG) protein-mediated H3K27me3 recruitment to genomic targets (Simon and Kingston, 2013). It has been shown that phosphorylation of Cbx2 is essential for its transcriptional repression of target genes, and that CK2-mediated phosphorylation inhibits Cbx2’s DNA-binding activity and transcriptional repression (Kawaguchi et al., 2017). Given that the expression of CK2 is virus-inducible in macrophages (GEO profile: GDS1271), we speculate that CK2-mediated phosphorylation inhibits recognition and binding to H3K27 in macrophages upon virus infection.

According to reported gene-profiling data, Cbx2 expression is downregulated in the brain (GEO: GSE44331) and in RAW264.7 macrophages (GEO: GSE81675), which is in agreement with our findings that Cbx2 expression decreases upon VSV infection. As a positive regulator of IFN-β transcription activation, the Cbx2 expression pattern differs from the expression pattern of other activators that are virus-inducible (Li et al., 2014a). This difference might be explained by a fine-tuning mechanism that serves to prevent superfluous IFN-β production. Proper termination of the antiviral effect is necessary to avoid excessive cytotoxicity and tissue injury. Therefore, Cbx2 down-regulation by VSV might constitute a feedback regulation mechanism in the antiviral response.

During virus infection, the host senses components derived from viruses and activates type I IFN response to eliminate viruses. On the other side, viruses have evolved kinds of ways to regulate and evade the host anti-viral immune response, such as sequestering anti-viral signaling transducers and effectors or targeting them for degradation. Furthermore, to establish virus infection-specific expression patterns of host cells for persistent infection or effective replication, virus may inhibit type I IFN response through epigenetic mechanisms (Garcia-Sastre, 2017). In this study, we observed that the expression of Cbx2, which is a coactivator of IFN-β, decreased during virus infection, although the underlying mechanism was unclear. Our study implies that virus may inhibit the host type I IFN response through blocking the positive epigenetic mechanism for transcription induction of INF-β.

In summary, we identified Cbx2 as a positive regulator for antiviral innate immunity. Our results suggest that Cbx2 promotes the production of IFN-β by associating with Jmjd3 and enhancing its recruitment to the *Ifnb* promoter. Our study provides mechanistic insight into epigenetic modifiers in the regulation of innate immunity.

## Materials and methods

### Animals

Cbx2^+/−^ mice were obtained from Jackson Laboratory (Stock No: 003478). The first 4 exons of Cbx2 were replaced via homologous recombination with a neomycin resistance cassette in reverse orientation. The strain was maintained by heterozygous mating for more than 2 years. Male C57BL/6J mice (six to eight weeks) were from Beijing Vital River Laboratory Animal Technology Co. (Beijing, China). All animals were maintained in specific-pathogen-free conditions. All animal experiments were carried out under the supervision of the Institutional Animal Care and Use Committee (IACUC) of the Institute of Basic Medical Sciences, Chinese Academy of Medical Sciences, Beijing, China.

### Plasmid constructs

A recombinant expression vector encoding N-Myc-tagged Cbx2 was constructed by PCR cloning into EX-Mm01605-M43 eukaryotic expression vector (Fulengene Genecopoeia). The IL-6 and IFN-β luciferase reporter plasmids have been described previously (Li et al., 2014a). Expression vectors encoding Flag-tagged Cbx2, Myc-tagged Jmjd3 and Myc-tagged IRF3 were constructed by PCR cloning into pcDNA3.1-Flag or pcDNA3.1-Myc eukaryotic expression vectors. Flag-tagged P65 and IRF3 expression vectors were constructed by PCR cloning into pcDNA3.1 eukaryotic expression vector (Invitrogen, Carlsbad, CA). All constructs were confirmed by DNA sequencing.

### Cells and cell transfection

HEK293T and RAW264.7 cell lines were from American Type Culture Collection. Thioglycolate-elicited mouse primary peritoneal macrophages were isolated and cultured, transfected with siRNA pool (10 nmol/L) through the use of Lipofectamine® RNAiMAX Reagent (Thermo Fisher Scientific). The siRNAs targeting mouse Cbx2 were as follow: CAAGUGCGGUCUCGGGCUA, GAGUCAAGUUCUCGUGUUU, UGGCAGAGCUCCAUCGUAC, CCGGGUAACUGUCUUGAAA (Designed by Dharmacon). For stable overexpression of Cbx2, we transfected cells expressing the IFN-β-Luc reporter gene (293T-IFN-β-Luc cells) or RAW264.7 cells with Myc-Cbx2 and selected the cells in G418 Sulfate (800 μg/mL; Gibco). For transient transfection of plasmids in HEK293T cells, Lipofectamine™ 3000 Reagent was used according to manufacturer’s instructions (Thermo Fisher Scientific). For transient transfection of plasmids in primary peritoneal macrophages, nuclear transfection was performed by using the AmaxaTMP3 Primary Cell 4D-nucleofectorTM X Kit (Lonza) according to the manufacturer’s instructions. All cells were cultured in endotoxin-free DMEM (Gibco), supplemented with 10% FBS (Gibco), 5 mg/mL penicillin (Gibco) and 10 mg/mL streptomycin (Gibco).

### Antibodies and reagents

Antibodies against Myc-tag (2272, 2276, 2040), Flag-tag (14793), phosphorylated IRF3 (Ser396) (#4947), phosphorylated NF-κB p65 (Ser536) (#3033), and Normal Rabbit IgG (#2729) were from Cell Signaling Technology. Antibodies against Cbx2 (LS-C81651, LS-C289259) were from LifeSpan BioSciences. Antibodies against Histone H3K27me3 (39155), Histone H3K9me3 (61013), and Histone H3K4me3 (61379) were from Active Motif. Antibodies against β-actin (M177-3) and GAPDH (M171-3) were from MBL. Protein G-agarose (20397) used for immunoprecipitation was from Pierce; ChIP Grade Protein G Magnetic Beads (#9006), and Cell Lysis Buffer (#9803) were from Cell Signaling Technology. Poly (I:C) was from Invitrogen. HSV was a gift from Prof. Q. Li (Chinese Academy of Sciences, Beijing, China), VSV was a gift from Prof. W. Pan (Second Military Medical University, Shanghai, China), and Sendai virus (SeV) was a gift from Prof. B. Sun (Chinese Academy of Sciences, Shanghai, China). HJP (Jmjd3 inhibitor) was a gift from X. Bin (Institute of Materia Medica, Chinese Academy of Sciences, Shanghai, China).

### Virus infection

For *in vitro* virus infection experiments, virus was added along with fresh medium to pre-treated cells for the indicated times at MOI of 10. For *in vivo* virus infection experiments, virus was injected via the tail vein.

### Quantitative RT-PCR

Total RNA was extracted with Trizol reagent (Ambion). ReverTra Ace® qPCR RT Master Mix with gDNA Remover (Toyobo) was used to synthesize cDNA. KOD SYBR® qPCR Mix (Toyobo) was used for qRT-PCR. Sample data were normalized to GAPDH expression. Primers for qPCR are listed in Table S1.

### ELISA

IFN-β and IL-6 levels in supernatants or sera were measured with mouse IFN-β ELISA kits (PBL Biomedical Laboratories).

### Immunoblot analysis

Cells and organs were lysed with RIPA Lysis Buffer (Merck Millipore), and extracted protein was homogenized by the BCA protein assay kit (Pierce). Immunoblot were performed with the indicated antibodies.

### VSV plaque assay

VSV titers of supernatants and tissue homogenate samples were determined by plaque assays on Vero cells. 100 µL samples (10-fold dilution) were inoculated onto confluent Vero cell monolayers for 1 h at 37 °C, 5% CO_2_, and washed by DMEM. And Vero cells were overlayed with a final concentration of 1% agar containing MEM, 1% FBS, 200 μg/mL diethylaminoethyl-dextran, and 0.008% for 48 h and 72 h. 0.008% neutral red was added to the cell monolayers, which were then incubated overnight, and plaques were counted. Viral burdens are reported as the log_10_ plaque forming units per mL (PFU/mL).

### Luciferase reporter assay

293T-IL-6-Luc cells or 293T-IFN-β-Luc cells were seeded in 96-well plates and then transfected with Cbx2 or empty plasmid and other signaling pathway-related molecular-expressing vectors as indicated, together with TK-Renilla-luciferase as a control. After 36 h of incubation, whole-cell lysates were collected for measurements of luciferase activity with the Dual-luciferase Reporter Assay System (Promega) according to the manufacturer’s instructions. The method has been described previously (Jiang et al., 2018).

### Chromatin immunoprecipitation (ChIP)

For ChIP assays, we used ChIP-IT® Express Chromatin Immunoprecipitation Kits (Active Motif) according to the manufacturer’s instructions. Primers used for ChIP quantification are listed in Table S2.

### Lung histology

Lung tissues from Cbx2^+/+^ mice and Cbx2^+/−^ mice were dissected and processed as described previously (Wang et al., 2017).

### Statistical analysis

Statistical significance between two groups was determined by the two tailed Student’s *t*-test. Differences were considered to be significant when *P* < 0.05. (*P < 0.05; **P < 0.01; ***P < 0.001). For mouse survival studies, Kaplan-Meier survival curves were generated and analyzed for statistical significance with GraphPad Prism 5.0.

## Electronic supplementary material

Below is the link to the electronic supplementary material.

Supplementary material 1 (PDF 348 kb)
