# Supplementary material for: Polycomb chromobox Cbx2 enhances antiviral innate immunity by promoting Jmjd3-mediated demethylation of H3K27 at the Ifnb promoter
Source: Protein Cell. 2018 Oct 24;10(4):285–94. doi: 10.1007/s13238-018-0581-0 (PMC6418077; doi:10.1007/s13238-018-0581-0)
Supplement: Supplementary file 1 — Supplementary material 1 (PDF 348 kb) [file 13238_2018_581_MOESM1_ESM.pdf]

Supplementary information:

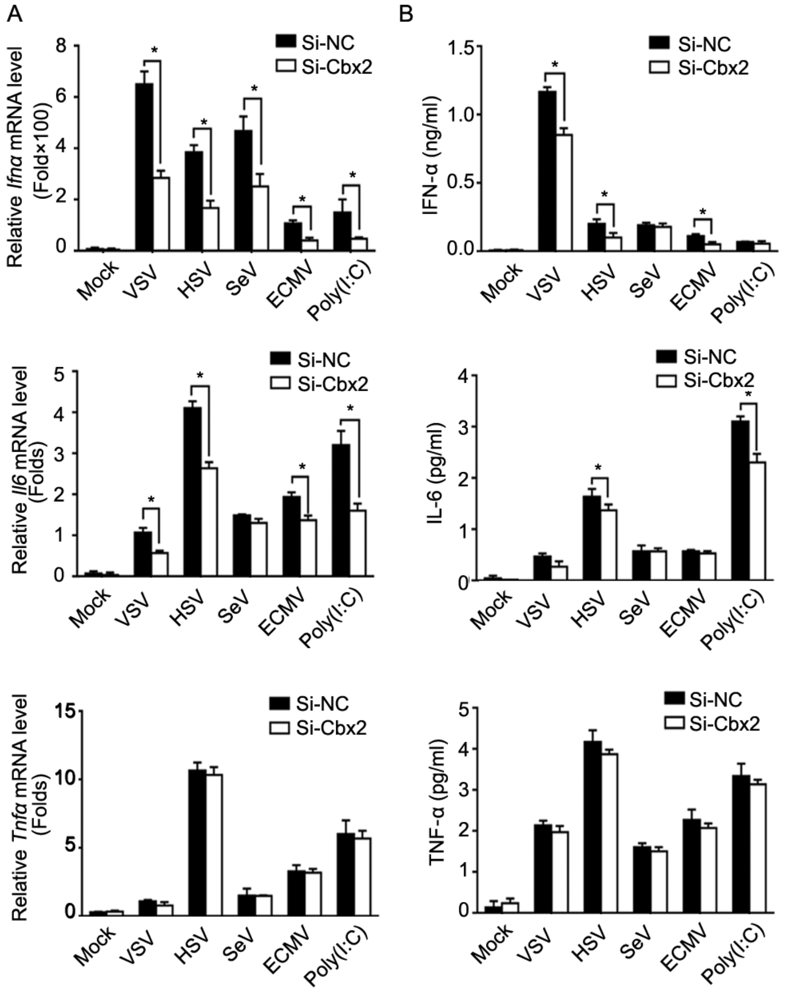

Figure S1. Cbx2 promotes virus-induced IFNα and Il-6 production in macrophages.

(A-B) Analysis the level of mRNA (A) and protein (B) of IFN-α, IL-6 and TNF-α in peritoneal macrophages silencing Cbx2 for 48 h by Q-PCR and ELISA. Cells were transfected with a control siRNA (Si-NC) or a Cbx2-targeting siRNA (Si-Cbx2) for 48h, and then infected with virus (MOI = 10) for 8 h (A-B). Data are representative of three independent experiments with similar results. * $p<0.05$.

Table S1, Q-PCR primers for gene expression analysis.

Genes	Sequence
Cbx2	F:5’-GGCTGGTCCTCCAAACACAA-3’<br>R:5’-CCCTGGGTCTCTTGCCTCT-3’
Cbx4	F:5’-AAGAAGCGGATACGCAAGGG-3’<br>R:5’-GGAGGAGTCTTGAAGCCCAG-3’
Cbx6	F:5’-GGAACCGGGTCATTGGGAAG-3’<br>R:5’-TAGAGCGCAAATGTGCCAAAC-3’
Cbx7	F:5’-TGCGGAAGGGCAAAGTTGAAT-3’<br>R:5’-ACAAGGCGAGGGTCCAAGA-3’
Cbx8	F:5’-ATTCGCAAAGGACGCATGGAA-3’<br>R:5’-CCTCGCTTTTTGGGGCCATA-3’
Hp1α	F:5’-GACAGGCGCATGGTTAAGG-3’<br>R:5’-CCTGGGCTTATTGTTTTCACCC-3’
Hp1β	F:5’-CCAAGGAAGCCAATGTCAAGT-3’<br>R:5’-GGAATGCCACGTTAGCCTTTC-3’
Hp1γ	F:5’-ACTGGACCGTCGTGTAGTGAA-3’<br>R:5’-GCCCCTTGGTTTGTCAGCA-3’
Ifnb	F:5’-CAGCTCCAAGAAAGGACGAAC-3’<br>R:5’-GGCAGTGTAACTCTTCTGCAT-3’
VSV	F:5’-ACGGCGTACTTCCAGATGG-3’<br>R:5’-CTCGGTTCAAGATCCAGGT-3’
actin	F:5’-AGGTCGGTGTGAACGGATTTG-3’<br>R:5’-TGTAGACCATGTAGTTGAGGTCA-3’

Table S2, Q-PCR primers for ChIP analysis of *Ifnb* promoter regions.

Regions	Sequence
up-1k	F:5’- AATAAAATATTTATAATACA-3’<br>R:5’-AAACAGAGTTTTTATTAA-3’
cds	F:5’- GCTATTACTGGAGGGTGC-3’<br>R:5’-AGTCCGCCTCTGATGCTTA-3’
up1k	F:5’-GCACAAGATCCTGGAATA-3’<br>R:5’-TGTGAGTGGGTAACAGAG-3’
up2k	F:5’-CGAGTTGACGGCACCCTA-3’<br>R:5’-TGCCACAGTGGGCAGATA-3’
up3k	F:5’-CCTCTGCTGGTGTCTTAG-3’<br>R:5’-AAGTAGGCTGAACAATCC-3’
up4k	F:5’-CATCACAGAGGGTTGCCTGT-3’<br>R:5’-CCACTGCCTCTCCTTCAGTG-3’
up5k	F:5’-AATGTCTGAGGCTACTGT-3’<br>R:5’-TGCATT CATACGCATCTA-3’
up6k	F:5’-GTAGATGTCAGCGGAGAA-3’<br>R:5’-ATCCTGGACAATGTAGCA-3’
up7k	F:5’-GAAGGTCCAGCAACAGGC-3’<br>R:5’-TGTCCATTGGTTCGGTGT-3’
up8k	F:5’-GAAGGTCCAGCAACAGGC-3’<br>R:5’-TGTCCATTGGTTCGGTGT-3’
up9k	F:5’ -CGTGGTGGGTACAAGCAG-3’<br>R:5’-CCTTCAGTTGGTGGTATT-3’
